# Deconstructing Ageism

**DOI:** 10.1093/geroni/igaa057.3212

**Published:** 2020-12-16

**Authors:** Laurinda Reynolds

**Affiliations:** American River College, Sacramento, California, United States

## Abstract

Fifty years of ageism research has identified the psychosocial subconstructs and consequences of ageism and produced over 30 published definitions. However, some educators still define ageism as age discrimination or prejudice against one age group by another age group. This oversimplification provides camouflage for insidious ageism, messages that are not recognized as harmful by their source or by the target of the ageism message. This type of ageism message cultivates implicit fears of aging and negative self-perceptions, and because they are not recognized, they undermine efforts to reframe aging. This presentation deconstructs ageism to make this extremely complex phenomenon clear without oversimplifying its nature or understating its consequences. It differentiates ageism from youthism through their developmental processes, persistence, and consequences. Knowledge will be integrated and synthesized across disciplines using a biopsychosocial lens. Examples of insidious ageism are presented to increase one’s capability to recognize and avoid insidious ageism.

